# Waddlia chondrophila: from a bovine abortigenic agent to a potential cause of human adverse pregnancy outcomes

**DOI:** 10.1099/jmm.0.002130

**Published:** 2026-03-26

**Authors:** Carole Kebbi-Beghdadi, David Longbottom, Hanna Marti, Gilbert Greub, on behalf of the ESCMID Study Group for Mycoplasma and Chlamydia Infections (ESGMAC)

**Affiliations:** 1 Institute of Microbiology, Lausanne University Hospital and University of Lausanne, Lausanne, Switzerland; 2 Moredun Research Institute, Penicuik, Midlothian, UK; 3 Institute of Veterinary Pathology, University of Zurich, Zurich, Switzerland

**Keywords:** abortion, miscarriage, bronchiolitis, intracellular, *Chlamydia*, Chlamydiota, Chlamydiales

## Abstract

*Waddlia chondrophila* is a strict intracellular bacterium belonging to the *Chlamydiales* order, which also encompasses important human and animal pathogens, including *Chlamydia trachomatis* and *Chlamydia psittaci*. Like all other members of the order, * W. chondrophila* displays a biphasic developmental cycle, alternating between infectious extracellular and replicative intracellular forms.

First isolated from a bovine aborted foetus, *W. chondrophila* was later demonstrated to be associated, in women, with adverse pregnancy outcomes, such as miscarriage. Furthermore, recent studies have demonstrated a high prevalence of anti-*W. chondrophila* antibodies in asymptomatic women who consulted doctors regarding infertility problems, and in the male patients of infertile couples. Additionally, a role for this bacterium in respiratory tract infections has been suggested, as it has also been recovered from respiratory samples.

Although the cause of miscarriage is not identified in ∼50% of cases, nowadays, no systematic diagnostic testing for the presence of *W. chondrophila* is routinely performed. Implementing large-scale testing in women with reproductive health issues would undoubtedly increase our knowledge of *W. chondrophila* prevalence and its implications for disease outcomes.

## Graphical Abstract

*Waddlia chondrophila* is an obligate intracellular bacterium that was first isolated from an aborted bovine foetus, but since then, it has been primarily found in different aquatic environments, including wells and hot water systems. In amoebae and other eukaryotic host cells, it has a biphasic developmental cycle, with an infectious extracellular form (elementary body) and a replicative intracellular form (reticulate body). *W. chondrophila* is a prevalent abortigenic agent in African cattle and is associated, in humans, with adverse pregnancy outcomes and respiratory infections. Its reservoir is probably mammals or amoebae, but the mode of transmission to humans remains unclear. It could be zoonotic (from companion animals or livestock, especially cattle), vector-borne (through arthropods) or water-borne (via infected amoebae).

## Historical perspective

The organism WSU 86-1044^T^ was first isolated from the pooled liver/lung homogenates of a first-trimester aborted bovine foetus from a 19-month-old primigravid Holstein cow at the Washington Animal Disease Diagnostic Laboratory in Pullman, WA, USA [1]. A combination of the organism’s morphology (size between ca. 0.2 and 0.5 µm) and its multiplication within the membrane-bound, cytoplasmic vacuoles that was observed by electron microscopy suggested that it had a close relationship with ehrlichiae or chlamydiae species. The sequencing of its 16S rRNA revealed an 84.5–85.3% sequence similarity with members of the order *Chlamydiales*. However, with less than 90% similarity, it did not fit into any of the families already present in this order. Therefore, in 1999, Rurangirwa *et al.* proposed that it should be classified in the *Chlamydiales* order as *Waddliaceae* fam. nov., *Waddlia chondrophila* gen. nov., sp. nov. [2].

*Waddlia* was named from the abbreviation WADDL (Washington Animal Disease Diagnostic-Laboratory) and chondrophila (which means ‘liking clumps’, from the Greek *chondros*=clump and *philos=*friendly to), and it was chosen to reference the association of the organism with cellular mitochondria [2].

## Clinical presentations

*W. chondrophila* has been isolated on two occasions from aborted bovine foetuses [1,3] and has been detected by PCR or immunocytochemistry in several aborted bovine placentas in Switzerland, Tunisia and South Africa [4,6]. In South Africa, * W. chondrophila* has also been detected in numerous ovine and caprine abortion cases [6]. In addition, in a pathogenesis study in which two bovine foetuses were experimentally infected with *W. chondrophila*, one of these foetuses died within 2 weeks [7]. More recently, in a *W. chondrophila* challenge study, one of nine pregnant heifers was observed to have pathogen-associated placental lesions. While the organism was detected in the diseased placenta, no abortion occurred [8]. These divergent results may be due to an inoculum that was too low or to any as-yet unknown confounding factor.

In humans, an association between miscarriage and the presence of anti-*W. chondrophila* antibodies has been observed in several large clinical studies [9,10]. It is noteworthy that most sero-epidemiological studies reported only IgG findings (when analysed, IgM positivity rates were low), suggesting that miscarriage is more likely due to reactivation of a chronic infection rather than a new acute infection acquired during pregnancy. However, a recent study conducted in Denmark on vaginal samples from 1,203 women experiencing spontaneous abortion could not confirm an association between the presence of *W. chondrophila* and miscarriage in this country [11]. This discrepant result may simply reflect a different epidemiology in Denmark, with less exposure to ruminants.

In addition, bacteria have been detected by PCR and immunohistochemistry from vaginal swab specimens or in the placentas of women experiencing miscarriage and preterm birth [12,13].

A limited association of anti-*W. chondrophila* antibodies and ectopic pregnancy has been highlighted by Hornung *et al*. [14]. In contrast, no association has been determined between *W. chondrophila* seroprevalence and preterm birth or tubal factor infertility [9,15].

Furthermore, *W. chondrophila* has also been detected in human respiratory samples by PCR, although its association with lower respiratory tract infections was not demonstrated [16,17].

## Microbial characteristics

### Phenotypic features

*W. chondrophila* are strict intracellular organisms with a biphasic developmental cycle. These coccoid, Gram-negative bacteria alternate between two developmental forms: the small and infectious elementary body (EB) (0.2–0.5 µm), which is non-replicative, and the larger reticulate body (RB) (0.5–1 µm), which is able to replicate but is non-infectious. When visualized by electron microscopy, EBs are darker (more electron-dense) than RBs, which is due to their condensed genetic material. EBs enter their host cell and reside in a bacteria-containing vacuole, called an inclusion, that rapidly evades the endocytic pathway and colocalizes first with host cell mitochondria before expressing endoplasmic reticulum (ER) markers such as calreticulin and Protein Disulfide Isomerase (PDI) [18]. At this point, EBs differentiate into RBs and start replication. After several rounds of replication, RBs asynchronously redifferentiate into EBs, which are released into the extracellular medium by cell lysis, ready to invade other host cells and begin a new infection cycle. When submitted to stressful conditions, bacteria can drastically enlarge and become aberrant bodies (ABs) [19]. Noteworthy, due to the presence of a class C β-lactamase-encoding gene, *W. chondrophila* is naturally resistant to penicillin and other β-lactam antibiotics. This explains why high doses of antibiotics are needed to induce the formation of ABs.

### Genotypic features

The *W. chondrophila* genome consists of a circular chromosome of 2.1 Mb and a circular plasmid of 15.6 kb, with a G+C content of 43.8 and 37.6 mol%, respectively [20]. There are ∼11 copies of the *W. chondrophila* plasmid per cell. The genome encompasses two rRNA operons, 37 tRNA genes and an estimated 1,934 protein-coding genes. In contrast to *Chlamydiaceae* family members, which almost completely lack repetitive elements, the genome also displays repeated sequences. Based on Clusters of Orthologous Genes (COG) annotation, 45 *W*. *chondrophila* proteins were assigned to the category ‘mobilome: prophages, transposons’, representing 2.3% of the total ORFs. Moreover, when compared with *Chlamydiaceae* bacteria, *W. chondrophila* exhibits enhanced metabolic capabilities for the synthesis of nucleotides, amino acids, lipids and other co-factors.

In addition, *W. chondrophila* encodes many virulence factors, including a functional type III secretion system (T3SS) [20] and a catalase [21], as well as a large variety of specific factors conferring resistance to host or environmental stresses.

## Laboratory confirmation and safety

### Specimen type

*W. chondrophila* was isolated on two occasions from aborted bovine foetuses [1,3]. It has also been retrieved from the diseased placenta of a heifer challenged with *W. chondrophila* [8]. In humans, it has been recovered from vaginal, placental and lower respiratory tract samples [12,13, 17].

### Laboratory confirmation

Currently, patient samples can be screened for the presence of *W. chondrophila* by quantitative PCR using primers specific to the bacterial 16S rRNA-encoding gene [16]. Additionally, the bacteria can be cultivated from human samples in amoebae or mammalian cells, which are then examined visually for the presence of the intracellular bacteria [22]. Moreover, Lienard *et al*. showed that anti-*W. chondrophila* antibodies can also be detected from patient sera by ELISA or micro-immunofluorescence (MIF) [23]. It is noteworthy that no serological cross-reaction is observed between *W. chondrophila* and *Chlamydia trachomatis* when using MIF and whole bacteria as antigens, as demonstrated by the very low positivity of sera towards both species [13,24].

One of the major limitations for broader *W. chondrophila* testing is the lack of commercially available CE-marked or FDA-approved diagnostic assays, i.e. lacl of assays compliant with European rules [CE = assays compliant with European rules [CE = “conformité européenne”] or with US regulations [FDA = Food and Drug Administration]. Without commercial tests and the marketing that typically accompanies them, laboratory managers currently have limited awareness of the importance of screening for *W. chondrophila*.

### Laboratory safety

As *W. chondrophila* is a human and animal pathogen, laboratory handling of the bacterium is performed under biosafety level 2 conditions. Laboratories must be equipped with biological safety cabinets and air filtration systems to limit exposure of people within the building and outside of it. These guidelines are suggested in all countries with laboratories that handle the culturing or isolation of *W. chondrophila* species.

## Treatment and resistance

### Resistance

*In vitro*, *W. chondrophila* is susceptible in amoebae to doxycycline and azithromycin but is resistant to fluoroquinolones, due to mutations in the GyrA and ParC quinolone resistance-determining regions, and to β-lactams [25]. The resistance to β-lactams is partially due to the presence of a β-lactamase (see above). In mammalian cells, treatment with penicillin derivatives, teicoplanin, vancomycin, fosfomycin and novobiocin induces the formation of ABs [19].

Of note, β-lactam antibiotics act on the peptidoglycan, which, in *W. chondrophila*, is pivotal to organize cell division.

All cases of *W. chondrophila* infection reported so far were documented retrospectively, and no data are available regarding *in vivo* antibiotic activity.

### Treatment

The current recommended treatment for infection with *W. chondrophila* is doxycycline (2×100 mg per day per os for 7–10 days) or azithromycin (500 mg per day per os for 3–5 days).

Based on (i) the high prevalence of *W. chondrophila* in the UK and in Switzerland, (ii) the strong serological association of * W. chondrophila* antibodies with miscarriage and (iii) the documentation of *W. chondrophila* in placentas in cases of miscarriage and preterm birth, we recommend the systematic screening of patients with adverse pregnancy outcomes, not only to enable treatment but also to avoid recurrent miscarriage.

## Pathogenic strategies

### Host range

*W. chondrophila* has been cultivated *in vitro* in a broad range of host cells, including protozoa, fish cells, insect cells and several types of mammalian cells from human, murine or simian origin [16,18, 22, 26,28]. Bacteria from the *Waddliaceae* family have, on some rare occasions, also been found in ticks [29,30] and, more recently, in the gut microbiome of swine lice [31].

### Vectors and sylvatic cycle

The main reservoir of *W. chondrophila* is likely mammals, and this hypothesis is supported by the very high *in vitro* permissivity of a large diversity of mammalian cell lines (see above). Amoebae are also a possible reservoir, since these bacteria were documented in well water in Spain and in hot water systems from non-domestic buildings in France [32]. However, this hypothesis was not supported by several studies that used amoebal coculture or amoebal enrichment to investigate water and wastewater treatment plants. In these studies, members of the *Parachlamydiaceae* and *Criblamydiaceae* families were detected far more frequently than *W. chondrophila* [33,35]. Given the ability of *W. chondrophila* to grow in insect cells and its detection in ticks by PCR, and recently by metagenomic sequencing in the gut microbiome of lice, arthropods should also be considered as possible vectors and reservoirs [28].

### Virulence factors

Like other members of the *Chlamydiales*, *W. chondrophila* possesses a T3SS, which enables the secretion of effector proteins into the host cell [20]. However, only a few of the identified T3SS effectors of *Chlamydiae* have homologues in the *W. chondrophila* genome. This diversity of effectors probably reflects the diversity of possible hosts. Although the *W. chondrophila* genome encodes no direct homologue of chlamydial inclusion membrane proteins (Incs), membrane proteins that decorate the inclusion membrane, 41 proteins with similar features, such as a bilobed hydrophobic domain, were identified by genome analysis [36]. Albeit little is known about the *W. chondrophila* effector repertoire, all proteins secreted by the T3SS, including Incs, most probably manipulate the bacterial environment to favour *W. chondrophil*a survival and replication [37,38].

In addition to T3SS effectors, examination of the *W. chondrophila* genome indicates the presence of virulence factors, such as the stress response proteins DnaK, GroEL and GroES, as well as the chromosome condensation protein HctA. A temperature-activated serine protease specific for unfolded proteins (HtrA), the tail-specific protease, which degrades p65 protein during the host cell immune response, the plasmid protein pGP6-D, which stimulates the host’s T-cell response, a *Chlamydia* protease-like activity factor and a catalase are also present [21,36].

Furthermore, the *W. chondrophila* genome encodes several proteins containing eukaryotic domains that likely mimic or interact with host cell proteins and can interfere with multiple biological processes. These eukaryotic domains include: (i) domains that target the nucleus and interfere with host transcriptional processes such as chromatin remodelling, DNA replication and repair, (ii) domains capable of manipulating the host ubiquitin system and (iii) domains involved in protein–protein interactions [36,39].

Overall, *W. chondrophila* is a highly effective intracellular parasite of eukaryotic cells, capable of recruiting mitochondria to the bacteria-containing vacuole, in order to exploit the ATP and lipids produced by this essential organelle [18]. This mitochondrial recruitment relies on microtubules and actin microfilaments and illustrates how effectors can hijack host cell functions for the bacterium’s benefit.

Together, T3SS effectors and proteins containing eukaryotic-like domains represent key strategies employed by *W. chondrophila* to manipulate the host cell.

## Epidemiology

### Transmission

The mode of transmission of *W. chondrophila* is still unclear. However, an association between domestic animals and *W. chondrophila* seropositivity has been reported, making zoonotic transmission likely [24]. In addition, given its ability to multiply in free-living amoebae and its presence in different water sources, water networks should also be considered as potential reservoirs and possible sources of infection for *W. chondrophila* [25,32, 40, 41]. Furthermore, since *W. chondrophila* has been found in ticks and lice and can multiply in insect cell lines, vector-borne transmission could also be possible [28,29, 31].

Human-to-human transmission by respiratory droplets has never been described, and no association has been observed between seropositivity and sexual exposure, making sexual transmission unlikely [42].

Within the *Chlamydiales* order, members of the *Waddliaceae* family display epidemiological patterns and reservoirs that differ markedly from those of other *Chlamydia*-related families, such as the *Parachlamydiaceae* and *Criblamydiaceae*, which are associated with amoebae, or the *Simkaniaceae* and *Rhabdochlamydiaceae*, which are linked to arthropods. Epidemiological data thus point to an association of *W. chondrophila* with mammals, supporting the view that these bacteria are well positioned to act as zoonotic agents and human pathogens.

### Infection

*Chlamydiales* bacteria use various mechanisms to attach to the host cell, including electrostatic interactions and specific ligand–receptor binding. In *W. chondrophila*, bacterial surface molecules, such as Hsp60 or OmcB, have been shown to be implicated in adhesion processes [43]. Moreover, *W. chondrophila* encodes other adhesins, including a Pmp-like autotransporter, as well as a family of OmpA-related proteins, whose large diversity is likely linked to the wide host range of these bacteria [43].

### Epidemiology

In Tunisia, *W. chondrophila* is one of the most prevalent abortigenic pathogens detected in cattle, with a seroprevalence greater than 10% [44,45]. Seroprevalence of *W. chondrophila* in asymptomatic women has been reported to be around 7% in England, 14% in Switzerland, 25% in Vietnam and 42% in Tunisia [9]. In asymptomatic men in Switzerland, seroprevalence has been reported to be around 20% [42].

Interestingly, these percentages are much higher when focusing on specific human populations, such as women with infertility in the Netherlands (46%) and the male patients of couples with infertility in Switzerland (58.3%) [46,47].

### Risk groups

Currently, nothing is known about risk groups. *W. chondrophila* was isolated from the placentas and vaginal swabs of women suffering from miscarriage [12,13]. It has also been recovered from respiratory samples from patients with community-acquired pneumonia and from children with bronchiolitis [16,17].

### Prevention

There are no current recommended measures for prevention, and no vaccine is available. However, since *W. chondrophila* could be transmitted from animals, by amoebae or by arthropods, reducing contact with these reservoirs and vectors might represent a suitable preventive strategy, especially during the first months of pregnancy. In addition, in cases of recurrent miscarriage of unknown aetiology, it may be worthwhile to test, before pregnancy, for the presence of these bacteria in the genital tract. In cases of a positive result, antibiotic treatment to eliminate the bacteria could be envisaged before a new pregnancy.

## Open questions

- What is the mode of transmission of *W. chondrophila*?
- What are the reservoirs and vectors of *W. chondrophila*? Could domestic animals or cattle be reservoirs of these bacteria?
- Is *W. chondrophila* a pathogen or a commensal bacterium?
- Is *W. chondrophila* truly pathogenic in humans, and is it a direct cause of miscarriage? Or could its apparent association with miscarriage simply reflect shared epidemiological patterns with an as-yet-unknown pathogen?
- Can *W. chondrophila* adopt a persistent stage in the genital tract of women and reactivate during pregnancy, causing miscarriage?
- What is the link, if any, between *W. chondrophila* and male infertility?
- Is co-infection with *C. trachomatis* possible? Are their inclusions fusogenic? What is the potential for genetic exchange between the two organisms?
- How does *W. chondrophila* interact with the immune system? What are the main cytokines that are at play during *W. chondrophila* infections?

## Abbreviations

AB: aberrant body
CE: Conformité européenne
COG: clusters of orthologous genes
EB: elementary body
ER: endoplasmic reticulum
FDA: Food and Drug administration
Incs: inclusion membrane proteins
MIF: micro-immunofluorescence
PDI: protein disulfide isomerase
RB: reticulate body
T3SS: type III secretion system
WADDL: Washington Animal Disease Diagnostic-Laboratory
